# Impact of pubertal timing on growth progression and final height in subjects affected by RASopathies

**DOI:** 10.3389/fendo.2024.1531545

**Published:** 2025-01-17

**Authors:** Federica Tamburrino, Laura Mazzanti, Dino Gibertoni, Concetta Schiavariello, Annamaria Perri, Eleonora Orlandini, Cesare Rossi, Marco Tartaglia, Marcello Lanari, Emanuela Scarano

**Affiliations:** ^1^ Pediatric Unit, IRCCS Azienda Ospedaliero-Universitaria di Bologna, Bologna, Italy; ^2^ Alma Mater University of Bologna, Bologna, Italy; ^3^ Statistics and Epidemiology Unit, IRCCS Azienda Ospedaliero-Universitaria di Bologna, Bologna, Italy; ^4^ Specialty School of Paediatrics, Alma Mater Studiorum, Università di Bologna, Bologna, Italy; ^5^ Medical Genetics Unit, IRCCS Azienda Ospedaliero-Universitaria di Bologna, Bologna, Italy; ^6^ Molecular Genetics and Functional Genomics, Ospedale Pediatrico Bambino Gesù, IRCCS, Rome, Italy

**Keywords:** Noonan syndrome, Mazzanti syndrome, RASopathies, puberty, growth hormone

## Abstract

**Background:**

RASopathies, including Noonan syndrome and related disorders, are multisystem conditions caused by mutations in various genes encoding proteins involved in the RAS/MAPK signaling pathway resulting in increased signal flow. They are clinically characterized by failure to thrive, facial dysmorphisms, congenital heart defects, lymphatic malformations, skeletal anomalies, and variable cognitive impairment, with variable prevalence in the different conditions and subtypes. Pubertal development, which affects growth and final height, is often delayed in Noonan syndrome patients, though not universally. This study aimed to evaluate the timing and progression of puberty and its impact on growth and final height in patients with RASopathies.

**Subjects and methods:**

A retrospective longitudinal study was conducted involving 103 patients with molecularly confirmed RASopathies. A subgroup of 40 patients who had completed pubertal development was analyzed. Anthropometric, hormonal (FSH, LH, estradiol/testosterone), and radiological data were collected.

**Results:**

Among the 40 patients who had completed puberty, 75% had a diagnosis of Noonan syndrome. The median age at pubertal onset was 11.8 years in males and 13.2 years in females. Delayed puberty was observed in 27.8% of patients, with a higher incidence in females. Median final height was significantly lower in those with delayed pubertal onset compared to those with normal development (p < 0.01). No significant differences in final height were observed between patients with growth hormone deficiency treated with growth hormone and those who were untreated.

**Conclusions:**

Delayed pubertal onset negatively impacts final height in patients with RASopathies, with inadequate pubertal catch-up growth being a common outcome. While most patients initiate puberty spontaneously, careful monitoring of growth and pubertal progression is crucial to optimize therapeutic interventions and improve final height outcomes.

## Introduction

RASopathies (Noonan syndrome (NS) and related disorders) are multisystemic conditions caused by mutations affecting multiple genes encoding proteins with a role in the RAS/MAPK signal transduction pathway (1). This family of developmental disorders includes NS (NS, MIM: PS163950) and an increasing number of related diseases that share major characteristics such as facial dysmorphism, postnatal reduced growth, congenital heart disease and hypertrophic cardiomyopathy, ectodermal features, skeletal anomalies, lymphatic malformations, variable cognitive involvement, cryptorchidism and susceptibility to certain malignancies, even though each characteristic has a variable prevalence in individual syndromes (2, 3).

Puberty is a process that marks the transition from childhood to adulthood, and influences the development of secondary sexual characteristics, reproductive organs, growth velocity and final height (FH), and self-esteem (4–6). In NS, pubertal development, though not in all subjects (7), is typically delayed by about 2 years, with a secondary peak growth rate lower than in the general population (GP), as usually happens in delayed puberty (8). Multiple cases of delayed puberty have been described (9, 10), including primary amenorrhea in females. Despite the delay, puberty begins spontaneously in most NS patients, indicating normal hypothalamic-pituitary function. Notably, in males with NS, fertility appears to be reduced, probably due to the high incidence (60–77%) of cryptorchidism (11, 12), and possibly because of primary alterations in the activity of Sertoli and Leydig cells (13, 14).

The age of onset and the duration of pubertal development significantly influence the growth spurt and near adult height in RASopathies, exacerbating the typical growth deficits associated with these conditions. In RASopathies, delayed pubertal development and inadequate pubertal catch-up growth could contribute to the observed reduced adult height.

Based on these considerations, the aim of this work was to evaluate the timing of the onset and progress of pubertal development, and its influence on growth and FH in a large group of patients affected by RASopathies.

## Patients and methods

A retrospective longitudinal study was performed involving 103 patients with clinical features fulfilling the criteria for RASopathies, and a clinical diagnosis confirmed by molecular analysis. We evaluated a subgroup of 40 subjects who had completed pubertal development. For 1 male patient, the starting age of puberty was not available. Patients were recruited at the Pediatric Rare Disease Outpatient Unit of IRCCS Azienda Ospedaliero-Universitaria di Bologna, Italy, between 2001 and 2023.

In most cases, molecular analyses were performed by massive parallel sequencing using a continuously updated panel of genes known to be implicated in these disorders. For a subset of patients, the molecular diagnosis had been achieved using targeted Sanger sequencing.

Pubertal stages were graded with the Tanner method (6). Clinical pubertal onset was defined as testicular volume ≥ 4 ml in boys and breast development (B2 Tanner stage) in females. At peri-pubertal age, we tested hypothalamic-pituitary-gonadal axis (follicle-stimulating hormone (FSH), luteinizing hormone (LH), estradiol/testosterone dosage). Luteinizing hormone, 17-β-estradiol and testosterone were considered detectable if ≥ 0.3 U/L, ≥ 15 pg/ml, and ≥ 0.2 ng/dl, respectively. The gonadotropin-releasing hormone agonist (GnRH) test was performed in patients with delayed puberty, in the laboratory of our university hospital clinic. All girls also underwent pelvic ultrasonography assessment. Bone age was determined using the Greulich and Pyle method.

In all patients cardiac function was periodically monitored by ECG and echocardiogram.

Among the different RASopathies in this cohort, we present, discuss, and compare the anthropometric, pubertal and hematochemical data collected from the NS and Mazzanti syndrome (*i.e.*, Noonan syndrome-like disorder with loose anagen hair NS/LAH, MIM: 607721) patients.

The study was conducted in accordance with the Declaration of Helsinki and Good Clinical Practice guidelines and was approved by the Emilia-Romagna AVEC ethics committee [internal code 279/2024/Oss/AOUBo].

### Statistical analysis

Height and body mass index (BMI, Kg/m^2^) were expressed as standard deviation scores (SDS) and referred for age- and sex-specific groups. Anthropometric measurements were compared to the standard growth curves for the general Italian population (15). Growth velocity SDS were calculated using Tanner charts (4, 5).

Comparisons between selected subgroups of patients were performed using nonparametric tests due to the limited sample size. When continuous variables were compared between independent groups, the Mann-Whitney test was used. Considering the limited cohort size and asymmetric distribution of most variables, the median was used to describe pubertal characteristics except when comparing the study population with the general population, for which mean ± standard devation was used.

All analyses were conducted with Stata v.17.0; p-values lower than 0.05 were considered statistically significant. Specifically, the pbreg procedure (16) was used for the Preece ± Baines estimation model. Comparisons between selected subgroups of patients were performed with nonparametric tests, due to the limited sample size. When continuous variables were compared between independent groups, the Mann-Whitney test was used.

## Results

Among the 103 patients in the study cohort, 40 subjects, 24 (60.0%) males and 16 (40.0%) females, had completed pubertal development. Clinically, 30 (75.0%) patients, had a diagnosis of NS; 7 (17.5%) subjects were diagnosed with NS/LAH, while the remaining 3 (7.5%) had a diagnosis of cardio-facio-cutaneous syndrome (CFCS; MIM: PS115150), Noonan syndrome with multiple lentigines (NSML; MIM: 151100), and Legius syndrome (MIM: 611431). Among the NS patients, 24 (80.0%) had *PTPN11* mutations, while each of the other genotypes accounted for less than 10%. All NS/LAH had a *SHOC2* mutation (**Tables 1A**, **B**). No patient had decompensated major heart disease.

**Table 1A:** Subgrouping of the studied RASopathy cohort by genotype.

	Entire cohort	
Genotype	n	%
Noonan syndrome	30	75.0
* PTPN11*	24	80.0
* KRAS*	2	6.7
* RAF1*	2	6.7
* RIT1*	1	3.3
* SOS1*	1	3.3
Noonan syndrome with multiple lentigines		
* PTPN11*	1	2.5
Cardiofaciocutaneous syndrome		
* BRAF*	1	2.5
Mazzanti syndrome		
* SHOC2*	7	17.5
Legius syndrome		
* SPRED1*	1	2.5
Total	40	100.0

n, number of patients.

**Table 1B:** Genetic variant classification in our cohort.

Case	Gene	Nucleotide change		Protein change
1	*PTPN11*	c.922 A>G		p.Asn308Asp
2	*PTPN11*	c.797G>C		p.Glu139Asp
3	*PTPN11*	c.236A>G		p.Gln79Arg
4	*PTPN11*	c.1403C>T		p.Thr468Met
5	*SPRED1*	c.1170dup		p.Cys391Leufs*4
6	*PTPN11*	c.922 A>G		p.Asn308Asp
7	*PTPN11*	c.922 A>G		p.Asn308Asp
8	*PTPN11*	c.922 A>G		p.Asn308Asp
9	*PTPN11*	c.1510A>G		p.Met504Val
10	*RAF1*	c.1472C>G		p.Thr491Arg
11	*RAF1*	c.1472C>G		p.Thr491Arg
12	*PTPN11*	c.1403C>T		p.Thr468Met
13	*PTPN11*	c.1504T>A		p.Ser502Thr
14	*SHOC2*	c.4A>G		p.Ser2Gly
15	*PTPN11*	c.922A>G		p.Asn308Asp
16	*SHOC2*	c.4A>G		p.Ser2Gly
17	*PTPN11*	c.205G>C		p.Glu69Gln
18	*PTPN11*	c.205G>C		p.Glu69Gln
19	*PTPN11*	c.1403C>T		p.Thr468Met
20	*BRAF*	c.770A>G		p.Gln257Arg
21	*KRAS*	c.458A>T		p.Asp153Val
22	*PTPN11*	c.1510A>G		p.Met504Val
23	*SHOC2*	c.4A>G		p.Ser2Gly
24	*PTPN11*	c.853T>C		p.Phe285Leu
25	*PTPN11*	c.923A>G		p.Asn308Ser
26	*RIT1*	c.67A>C		p.Lys23GLn
27	*SHOC2*	c.4A>G		p.Ser2Gly
28	*SHOC2*	c.4A>G		p.Ser2Gly
29	*SHOC2*	c.4A>G		p.Ser2Gly
30	*PTPN11*	c.922 A>G		p.Asn308Asp
31	*PTPN11*	c.922 A>G		p.Asn308Asp
32	*PTPN11*	c.124A>G		p.Thr42Ala
33	*PTPN11*	c.178G>A		p.Gly60Ser
34	*PTPN11*	c.188A>G		p.Tyr63Cys
35	*SHOC2*	c.4A>G		p.Ser2Gly
36	*SOS1*	c.797C>A		p.Thr266Lys
37	*KRAS*	c.178G>A		p.Gly60Ser
38	*PTPN11*	c.188A>G		p.Tyr63Cys
39	*PTPN11*	c.922 A>G		p.Asn308Asp
40	*PTPN11*	c.236A>G		p.Gln79Arg

Twenty-eight patients reached FH, 23 of whom had been treated with growth hormone (GH) therapy due to growth hormone deficiency (GHD).

### Pubertal timing, profiling, and duration of puberty

**Figure 1:**
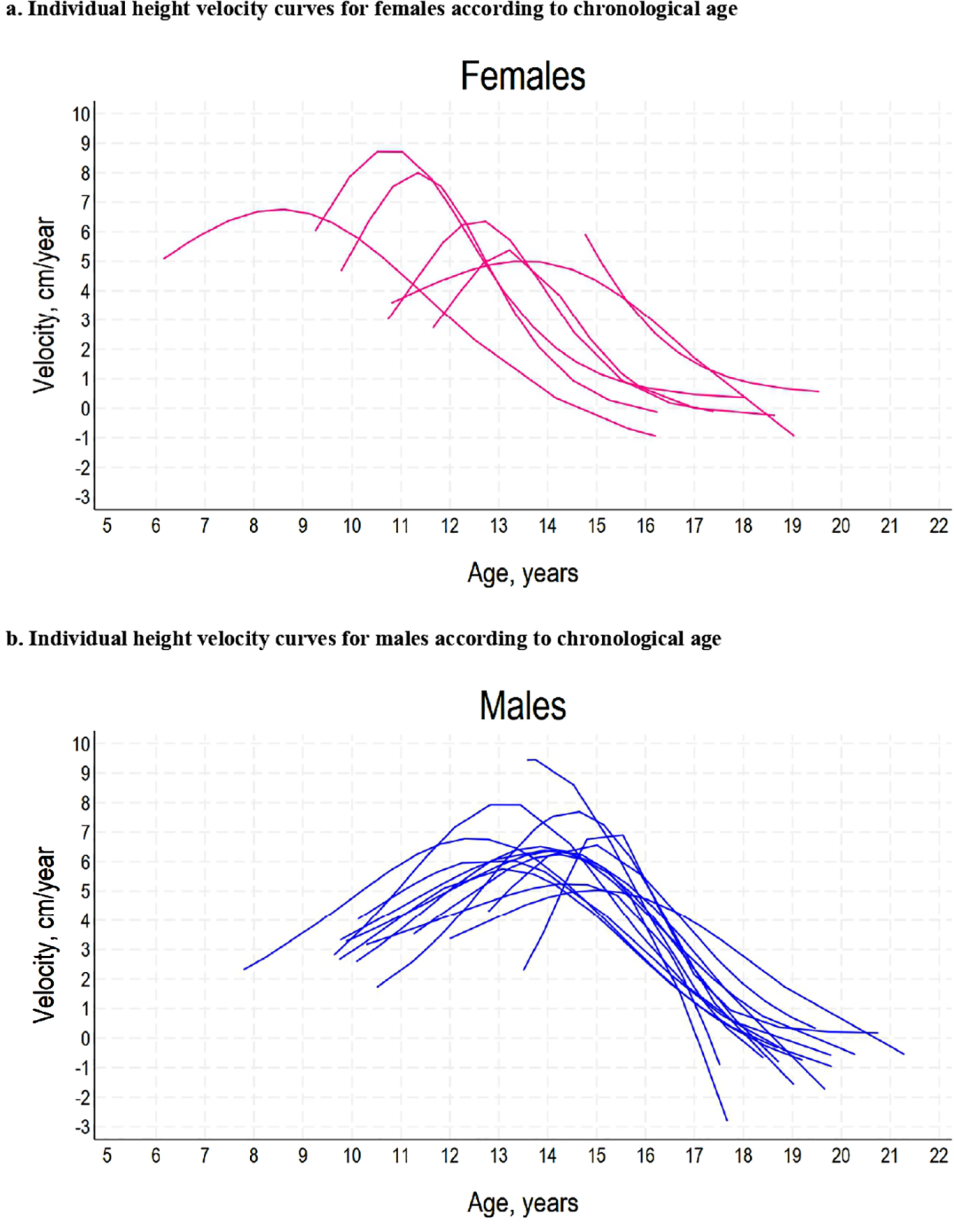
**(A)** Individual height velocity curves for females according to chronological age. **(B)** Individual height velocity curves for males according to chronological age.

In the cohort studied, mean age at onset of puberty was 11.96 ± 0.17 in males and 12.92 ± 0.25 in females, reaching a median peak height velocity (PHV) of 8.30 ± 0.22 cm/year and 5.89 ± 0.20 cm/year, respectively (**Figure 1**).

Mean spurt gain was 25.98 ± 0.91 cm in males and 13.80 ± 1.25 cm in females. A comparison between the present cohort and the general population (GP) referring to Tanner standards (17, 18) is reported in **Table 2**. Delayed puberty was observed in 10 out of 39 (25.6%) patients, 9 of whom were females. Differences in pubertal data between genotypes were not significant (**Table 3**).

**Table 2:** Comparison of pubertal data of the study cohort with the general population (GP).

	Study cohort (mean ± SDS)	GP (mean ± SDS)	test; p-value
Age at onset of puberty (years)			
M	11.96 ± 0.17	12.05 ± 0.85	-0.5; 0.586
F	12.92 ± 0.25	10.3 ± 0.95	10.7; <0.001
Age at PHV (years)			
M	14.28 ± 0.18	13.91 ± 0.84	2.1; 0.035
F	12.86 ± 0.23	11.89 ± 0.90	4.2; <0.001
PHV (cm/years)			
M	8.30 ± 0.22	8.80 ± 1.05	-2.3; 0.021
F	5.89 ± 0.20	8.13 ± 0.78	-11.1; <0.001
Spurt gain (cm)			
M	25.98 ± 0.91	27.56 ± 3.54	-1.7; 0.084
F	13.80 ± 1.25	25.25 ± 4.14	-9.2; <0.001

Data referring to the GP are from (5).

PHV, peak of height velocity.

**Table 3:** Comparison of pubertal data by genotype (*PTPN11* vs *SHOC2* patients).

PUBERTAL CLINICAL PARAMETERS	*PTPN11* (n=25) Median [IQR]	*SHOC2* (n=7) Median [IQR]	p-value
Age at puberty onset (yrs)	12.33 [11.32-13.25]	12.73 [11.42-14.07]	0.469
Height at puberty (cm)	135.55 [131.95-140.8]	133.9 [129.5-143.5]	0.686
Spurt duration (yrs)	6.64 [5.43-7.75]	5.665 [3.23-7.995]	0.764
PHV (cm)	7.86 [6.60-8.33]	6.19 [5.43-6.34]	0.070
Spurt gain (cm)	20.6 [19.6-26.2]	14.25 [3.40-29.95]	0.531
Age at menarche (yrs)	14.45 [12.28-16.40]	19.4 [14.64-19.41]	0.066
Final height (cm)	159.3 [151.7-165.8]	149.5 [148.4-158.4]	0.256

PHV, peak of height velocity.

Twenty-four male patients had cryptorchidism. Age at menarche was 15.48 (IQR 13.66-18-53) years.

Final height was reached by 28/40 patients (70.0%), of whom 13 were female and 15 were male, at a median age of 18.11 years [IQR 16.50-19.07] in females and 18.72 [IQR 18.14-19.80] in males.

**Figure 2:**
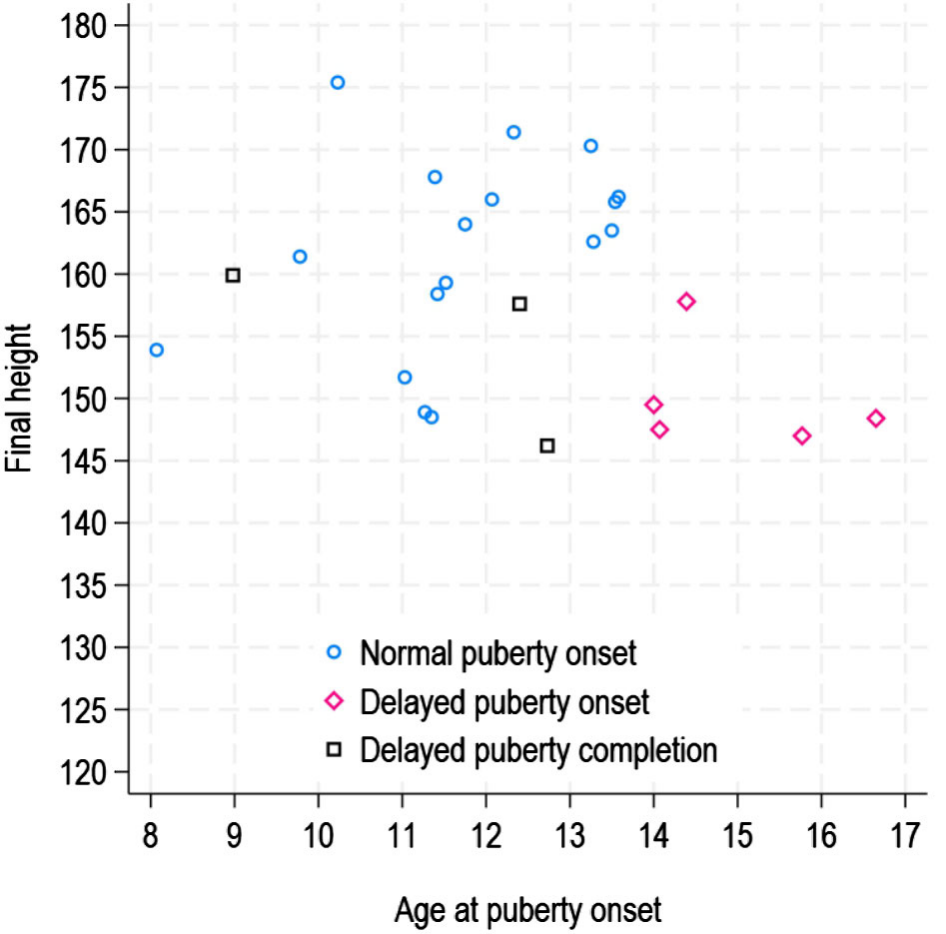
Final height according to pubertal timing.

In **Figure 2**, pubertal timing data are reported for the patients who reached FH. Analyzing the FH values in relation to the timing of the onset and completion of puberty, significant differences were observed (χ² = 9.04, p < 0.01): subjects with delayed onset of puberty had median FH values of 148.4 cm (147.0 - 157.8), which were lower than those of subjects with normal pubertal onset [163.1 cm (148.5 - 175.4)] or with delayed completion of puberty [157.6 cm (146.2 - 159.9)].

At the first evaluation, height was significantly lower in patients with GH deficit than in patients without GH deficit. No significant differences were observed between treated or not treated patients in terms of pubertal onset, spurt duration, spurt gain or FH (**Table 4**). No significant differences were seen between *PTPN11* and *SHOC2* regarding age of pubertal onset, duration of the growth spurt, or spurt gain.

**Table 4:** Comparison of clinical and pubertal features by GH-therapy in the studied RASopathy cohort.

PARAMETERS	GH-treated patients (23 pts) Median [IQR]	Untreated GH patients (5 pts) Median [IQR]	p-value
Age at 1st evaluation (yrs)	5.26 [3.09, 9.25]	6.09 [4.85, 7.66]	0.976
Height at 1st evaluation SDS	-2.9 [-3.5, -2.1]	-1.2 [-2.0, -0.4]	0.032
Age at PHV (yrs)	14.2 [12.6, 15.4]	13.4 [12.7-13.9]	0.529
Height at PHV (cm)	145.9 [141.1-154.1]	152.2 [135.6-154.4]	0.787
Spurt duration (yrs)	6.2 [5.4, 7.3]	8.1 [3.8-9.6]	0.407
Spurt gain (cm)	21.5 [15.7, 25.4]	25.7 [16.9-33.4]	0.286
Final height (cm)	157.8 [148.5-165.8]	159.9 [154.4-164.0]	0.653
Final height SDS	-2.2 [-2.6, -1.5]	-1.7 [-2.3, -0.7]	0.286
Target height (cm)	166.5 [160.0, 171.0]	161.75 [158.0-167.5]	0.417
Pubertal timing, n (%)			0.118 ^
◼ normal	15 (71.4%)	3 (60.0%)	
◼ delayed onset	5 (23.8%)	0	
◼ delayed completion	1 (4.8%)	2 (40.0%)	

GH, growth hormone; pts, patients; PHV, peak of height velocity, yrs, years.

Mann-Whitney test except ^ (Fisher’s exact test).

### Gonadal function

Analyzing the gonadal function biochemical parameters at the beginning of puberty, in females the mean age at which LH became detectable (≥0.3 U/L) was 15.74 ± 3.18 years, the median age of LH/FSH > 1 was 15.79 (IQR=12.70), and the age at which age 17-β-estradiol became detectable (≥ 15 pg/ml) was 13.6 ± 2.9 years. In males, mean age at dosable LH (≥ 0.3 U/L) was 13.97 ± 2.51 years, median age of LH/FSH > 1 was 15.54 (IQR = 4.99), mean age at dosable testosterone (≥ 0.2 ng/ml) was 13.20 ± 1.62 years. In 8 patients (4 females and 4 males) with suspected pubertal delay, the LHRH test was performed, which ruled out conditions of hypogonadism.

### Fertility

In this case series, we did not collect data on hormonal parameters to predict gonadal reserve. Nevertheless, 3 subjects who did not experience delayed puberty had offspring: 2 males carrying heterozygous *PTPN11* mutations, 1 of them with cryptorchidism, had children by means of assisted reproductive techniques, and 1 female (*RAF1* mutation) had a spontaneously conceived pregnancy.

## Discussion

Puberty is a complex and crucial process that influences the overall development of every individual; it involves physical changes, reproductive maturity, and neurocognitive and psychological shifts. The timing of pubertal development can vary greatly among individuals, influenced by genetic, nutritional, environmental, and socioeconomic factors. Understanding how puberty affects growth velocity (GV) and FH is crucial for assessing and managing conditions related to growth disorders.

The impact of puberty on GV and FH is significant, especially in conditions characterized by short stature and alterations in pubertal timing, including the RASopathies. In NS, pubertal development is frequently delayed, although puberty begins spontaneously in most NS patients, indicating normal hypothalamic-pituitary function (6, 7, 19). Furthermore, the hypothesis that lower body weight could also influence puberty timing has been suggested (20).

In a cohort of patients clinically diagnosed with NS, Romano et al. (7) reported that 35% of males started puberty later than 13.5 years, and 44% of females later than 13 years, and that SDS height at pubertal onset was highly correlated with near adult SDS height. Additionally, the duration of puberty was highly correlated with pubertal height gain in centimeters for males and females.

In our previously reported subgroup of 12 patients treated with GH who reached adult height, pubertal growth showed a lowered peak and a pubertal onset delay of approximately 6 months compared to the GP (21).

In a large cohort of 133 Brazilian individuals molecularly diagnosed with NS, Rezende et al. (20) observed that girls experienced delayed puberty more frequently than boys (49.1% *vs* 27.9%) at a median age of 11.9 ± 1.9 years in girls and 12.5 ± 1.7 years in boys. These ages were significantly later compared to the general Brazilian population, but earlier than reported by Shaw et al. (9) and Romano et al. (7), possibly reflecting different genetic backgrounds among populations. This data was corroborated by Libraro et al. (22) who, in a retrospective, multicenter Italian study including 228 subjects with a molecular diagnosis of NS, reported delayed puberty in 45% of females and 10% of males, with a mean age at onset of puberty of 12.1 ± 2.3 years in females, while males started puberty at 12.1 ± 1.3 years.

In line with previously published findings (4, 5), in our cohort females exhibited a significant delay in the onset of puberty, along with a reduction in PHV and spurt gain compared to the GP.

The discrepancy between the onset of Tanner stage B2 and the detectability of LH could be attributed to the pulsatile nature of LH secretion. During Tanner stage 2, LH is often detectable only during nighttime pulses, which may not always be captured depending on the timing of the tests. However, the significant delta support a potential delay in pubertal progression that warrants further evaluation to rule out any underlying pathological factors.

Moreover, the difference in age of menarche onset compared to the GP appeared relevant. By contrast, males showed only a mild delay in the age at which they reached PHV and a slight reduction in growth spurt gain.

No significant differences between *PTPN11* and *SHOC2* regarding age of pubertal onset, duration of the growth spurt, and spurt gain were observed. The age of menarche in *SHOC2* patients is markedly delayed compared to the GP and to *PTPN11*, although it does not reach statistical significance (**Table 3**).

The subjects who experienced delayed onset of puberty were disadvantaged in terms of FH when compared to those with either normal onset or delayed completion of puberty [3]. The results emphasize that a delay in the start of puberty had a negative impact on the amount of height gained during this critical growth period. Essentially, the timing of pubertal onset plays a crucial role in determining FH outcome, and delayed onset, as opposed to delayed completion, seems to have more adverse consequences.

Analyzing the effect of pubertal development in the 23 subjects with GH deficiency treated with GH, we did not observe any significant difference compared to untreated patients regarding the duration of the growth spurt, spurt gain, and FH, confirming the positive effect of GH therapy in those with a deficiency, as previously reported by Tamburrino et al. (21).

Female fertility appears to remain preserved in individuals with NS. However, males with NS often experienced reduced fertility, primarily due to the high prevalence of cryptorchidism (60-77%) (11, 12).

Abnormalities in gonadal function among males with NS are frequently under-characterized and poorly described. The natural history of certain aspects of NS in adulthood, including male fertility, remains poorly understood, and the exact prevalence of infertility in adult males with NS is unknown.

Studies on gonadal function in NS males, spanning both the prepubertal period (23) and adulthood (24–26), suggest the primary involvement of Sertoli cells. Moniez et al. (14) showed normal testosterone levels in NS pubertal boys, suggesting a normal Leydig cell function but, in contrast, significantly lower levels of Anti-Müllerian hormone (AMH) and inhibin B compared with the GP, suggesting a Sertoli cell dysfunction, in particular in *PTPN11* patients. Instead, Ankarberg-Lindgren et al. (13) reported dysfunction in both Sertoli and Leydig cells in 12 NS males, resulting in the progressive impairment of reproductive hormone levels in adults. Four individuals exhibited delayed puberty and reduced testicular volume during puberty, though only two had reduced testicular volume in adulthood. In adults, levels of LH, FSH, testosterone, and estrogen were elevated, while AMH and inhibin B levels were comparable to controls. There was no significant difference in hormone levels between those with or without cryptorchidism. Thus, it is hypothesized that reproductive dysfunction in NS males primarily stems from intrinsic alterations in Sertoli and Leydig cell activity, rather than solely from cryptorchidism.

Although based on a small number of patients, the present case series allows us to confirm what has already been reported in the literature regarding fertility in NS. In fact, one female had a spontaneous pregnancy, and two males, one of whom had cryptorchidism, needed to resort to assisted reproductive techniques.

The issue of fertility in NS needs to be explored further in a larger number of patients and in collaboration with reproduction specialists. It will be necessary to conduct multicenter studies to analyze the progression of puberty and the effects of pubertal timing on growth and FH in patients with RASopathies.

### Limitations

Several limitations should be considered when interpreting this study’s findings. The small sample size, particularly in subgroups such as patients with GH deficiency and specific RASopathies, limits the generalizability of results. Larger, more diverse cohorts are needed to strengthen conclusions on the impact of pubertal timing on growth and final height (FH). The retrospective nature of some data may introduce biases in the accuracy of pubertal onset and duration. Additionally, while females with NS show significant delays in pubertal onset, the underlying mechanisms and long-term effects on growth and reproductive health require further investigation. Fertility in females, though largely preserved, needs more thorough exploration. Gonadal function, especially in males with cryptorchidism, remains underexplored, and the prevalence and mechanisms of infertility in adult males with NS are unclear. Further studies with larger samples and long-term follow-up are needed to better understand these aspects.

## Conclusions

In conclusion, patients with RASopathies are known to exhibit short stature. Among them, those with delayed pubertal onset face an additional disadvantage compared to individuals with normal pubertal onset, significantly impacting growth and FH. Multicenter studies will be valuable for investigating the progression of puberty and the impact of its timing on growth and FH in patients with RASopathies. Expanding the patient cohort could also provide valuable insights into the role of genotype in shaping pubertal development and reproductive health.

## Data Availability

Datasets are available on request. The raw data supporting the conclusions of this article will be made available by the authors, without undue reservation.

## References

[1] Tartaglia M Aoki Y Gelb BD. The molecular genetics of RASopathies: An update on novel disease genes and new disorders. Am J Med Genet C Semin Med Genet. (2022) 190:425–39. doi: 10.1002/ajmg.c.32012 PMC10100036 36394128

[2] Tartaglia M Gelb BD Zenker M. Noonan syndrome and clinically related disorders. Best Pract Res Clin Endocrinol Metab. (2011) 25:161–79. doi: 10.1016/j.beem.2010.09.002 PMC3058199 21396583

[3] Rauen KA. The RASopathies. Annu Rev Genomics Hum Genet. (2013) 14:355–69. doi: 10.1146/annurev-genom-091212-153523 PMC4115674 23875798

[4] Tanner JM Whitehouse RH Takaishi M. Standards from birth to maturity for height, weight, height velocity, and weight velocity: British children, 1965. I. Arch Dis Child. (1966) 41:454–71. doi: 10.1136/adc.41.219.454 PMC2019592 5957718

[5] Tanner JM Whitehouse RH Takaishi M. Standards from birth to maturity for height, weight, height velocity, and weight velocity: British children, 1965. II. Arch Dis Child. (1966) 41:613–35. doi: 10.1136/adc.41.220.613 PMC2019692 5927918

[6] Tanner JM Whitehouse RH Marubini E Resele LF. The adolescent growth spurt of boys and girls of the Harpenden growth study. Ann Hum Biol. (1976) 3:109–26. doi: 10.1080/03014467600001231 1275435

[7] Romano AA Dana K Bakker B Davis DA Hunold JJ Jacobs J. Growth response, near-adult height, and patterns of growth and puberty in patients with Noonan syndrome treated with growth hormone. J Clin Endocrinol Metab. (2009) 94:2338–44. doi: 10.1210/jc.2008-2094 19401366

[8] Otten BJ Noordam C. Growth in noonan syndrome. Horm Res. (2009) 72 Suppl 2:31–5. doi: 10.1159/000243776 20029234

[9] Shaw AC Kalidas K Crosby AH Jeffery S Patton MA. The natural history of Noonan syndrome: a long-term follow-up study. Arch Dis Child. (2007) 92:128–32. doi: 10.1136/adc.2006.104547 PMC2083343 16990350

[10] Patti G Scaglione M Maiorano NG Rosti G Divizia MT Camia T. Abnormalities of pubertal development and gonadal function in Noonan syndrome. Front Endocrinol (Lausanne). (2023) 14:1213098. doi: 10.3389/fendo.2023.1213098 37576960 PMC10422880

[11] Noonan JA. Noonan syndrome and related disorders: alterations in growth and puberty. Rev Endocr Metab Disord. (2006) 7:251–5. doi: 10.1007/s11154-006-9021-1 PMC1894828 17177115

[12] Allanson JE. Noonan syndrome. Am J Med Genet C Semin Med Genet. (2007) 145C:274–9. doi: 10.1002/ajmg.c.30138 17639592

[13] Ankarberg-Lindgren C Westphal O Dahlgren J. Testicular size development and reproductive hormones in boys and adult males with Noonan syndrome: a longitudinal study. Eur J Endocrinol. (2011) 165:137–44. doi: 10.1530/EJE-11-0092 21551165

[14] Moniez S Pienkowski C Lepage B Hamdi S Daudin M Oliver I. Noonan syndrome males display Sertoli cell-specific primary testicular insufficiency. Eur J Endocrinol. (2018) 179:409–18. doi: 10.1530/EJE-18-0582 30325180

[15] Cacciari E Milani S Balsamo A Spada E Bona G Cavallo L. Italian cross-sectional growth charts for height, weight and BMI (2 to 20 yr). J Endocrinol Invest. (2006) 29:581–93. doi: 10.1007/BF03344156 16957405

[16] Sayers A Baines M Tilling K. (2013) A new family of mathematical models describing the human growth curve—Erratum: Direct calculation of peak height velocity, age at take-off and associated quantities. Annals of Human Biology. 40 (3):298–299. doi: 10.3109/03014460.2013.772655 23461542

[17] Marshall WA Tanner JM. Variations in pattern of pubertal changes in girls. Arch Dis Child. (1969) 44:291–303. doi: 10.1136/adc.44.235.291 5785179 PMC2020314

[18] Marshall WA Tanner JM. Variations in the pattern of pubertal changes in boys. Arch Dis Child. (1970) 45:13–23. doi: 10.1136/adc.45.239.13 5440182 PMC2020414

[19] Ranke MB Heidemann P Knupfer C Enders H Schmaltz AA Bierich JR. Noonan syndrome: growth and clinical manifestations in 144 cases. Eur J Pediatr. (1988) 148:220–27. doi: 10.1007/BF00441408 3215198

[20] Rezende RC Noronha R Keselman A Quedas EPS Dantas NCB Andrade NLM. Delayed puberty phenotype observed in noonan syndrome is more pronounced in girls than boys. Horm Res Pediatr. (2022) 95:51–61. doi: 10.1159/000522670 35176743

[21] Tamburrino F Gibertoni D Rossi C Scarano E Perri A Montanari F. Response to long-term growth hormone therapy in patients affected by RASopathies and growth hormone deficiency: Patterns of growth, puberty and final height data. Am J Med Genet A. (2015) 167A:2786–94. doi: 10.1002/ajmg.a.37260 26227443

[22] Libraro A D’Ascanio V Cappa M Chiarito M Digilio MC Einaudi S. Growth in children with noonan syndrome and effects of growth hormone treatment on adult height. Front Endocrinol (Lausanne). (2021) 12:761171. doi: 10.3389/fendo.2021.761171 35002956 PMC8730290

[23] Theintz G Savage MO. Growth and pubertal development in five boys with Noonan’s syndrome. Arch Dis Child. (1982) 57:13–7. PMC2863270 6121534

[24] Sinisi AA Criscuolo T Maresca F Quarto C Di Finizio B Bellastella A. Endocrine profile in Noonan’s syndrome. Minerva Endocrinol. (1987) 12:13–7. 3108642

[25] Elsawi MM Pryor JP Klufio G Barnes C Patton MA. Genital tract function in men with Noonan syndrome. J Med Genet. (1994) 31:468–70. doi: 10.1136/jmg.31.6.468 PMC1049925 7915331

[26] Marcus KA Sweep CG van der Burgt I Noordam C. Impaired Sertoli cell function in males diagnosed with Noonan syndrome. J Pediatr Endocrinol Metab. (2008) 21:1079–84. doi: 10.1515/jpem.2008.21.11.1079 19189703

